# Viburnum Prunifolium

**Published:** 1879-04-20

**Authors:** G. S. Glazener

**Affiliations:** S. C.


### *VIBURNUM PRUNIFOLIUM.*

BY G. S. GLAZENER, M.D., OF S. C.

I have had much and varied success in the use of viburnum. At first I used the viburnum in cases of abortions and miscarriages, and always with success, if the membranes had not been ruptured previous to my visit, as the following cases will show; representing also many others.

On the 28th of November last, I was called to see Mrs. W——; found her having active labor pains every five minutes. She was six months pregnant, the membranes not ruptured. I gave one teaspoonful tincture viburnum. In twenty minutes repeated the dose. In ten minutes more pain stopped and the woman asleep. I ordered medicine repeated upon least return of pain, and for a few days to take a dose every four or six hours. Child carried to full term.

The following day I was called to see Mrs. B——, in labor at full time. Child delivered, and followed by hemorrhage. I used means to stop that immediately, and in one hour left the woman doing well. In a few hours was recalled and was urged to give "something to stop these severe and unbearable after-pains." Now I immediately reasoned in this way: If viburnum pays its attention directly to the womb to stop pain in an abortion, why will it not stop pain after delivery? Two doses given as above stopped the pain, and ordered to be repeated if necessary. The case had no further trouble.

Now can it be objected that it is not best to stop after-pains? Do after-pains protect parturient woman in any way? If it is nature's own work for her protection, then why do some women never have after-pains? With viburnum, I think I can safely say, let no woman suffer with them.

Recently I have prescribed the viburnum in menorrhagia, lessening and controling the flow; and in dysmenorrhœa, controling the pain at will; and in one case of uterine neuralgia I used it with marked and almost certain effect. This opens up a wide field in practice, and I would like to hear of the experience of "our medical friends" generally, through the Record, in the use of this agent.

